# Clinicopathological and Prognostic Characteristics of Hepatoid Adenocarcinoma of the Stomach

**DOI:** 10.1155/2014/140587

**Published:** 2014-02-11

**Authors:** Jinlin Yang, Rui Wang, Wenyan Zhang, Wen Zhuang, Mojin Wang, Chengwei Tang

**Affiliations:** ^1^Department of Gastroenterology, West China Hospital, Sichuan University, Chengdu 610041, China; ^2^Department of Pathology, West China Hospital, Sichuan University, Chengdu 610041, China; ^3^Department of Gastrointestinal Surgery, West China Hospital, Sichuan University, Chengdu 610041, China

## Abstract

The present study was undertaken to clarify the association of the clinicopathological features of hepatoid adenocarcinoma (HAC) in the stomach, a special kind of carcinoma that histologically resembled hepatocellular carcinoma (HCC) and is characterized by large amounts of **α**-fetoprotein (AFP) in serum, with the clinical prognosis. We collected the data of the clinicopathological features and the follow-up information from a total of 31 HACs from January 2005 to December 2012 in our hospital. High lymphatic (54.8%) and distant (25.8%) metastasis rates before surgery, large proportion of advanced HACs (71.0%) at admission, short median overall survival time (6 months), and low three-year survival rate (22.6%) suggested that HAC in the stomach was an aggressive disease, resulting in a poor prognosis. And pTNM stages, immunohistochemical staining of AFP, CEA, CK7, and CK20 had statistically relation with the survival as the independent risk factors, *P* < 0.05. Therefore, early and clear differentiation of HAC from cancerous or noncancerous conditions with AFP elevation and assessment of high risk patients by histopathology may improve the clinical prognosis.

## 1. Introduction

Hepatoid adenocarcinoma (HAC), a special kind of carcinoma that histologically resembles hepatocellular carcinoma (HCC) and characterized by large amounts of *α*-fetoprotein (AFP) in serum, has been described in various organs. Stomach is one of the organs in which HAC has been most commonly identified. Remarkable characteristics of HAC in the stomach are its extensive vascular and lymphatic invasions and frequent hepatic metastases, resulting in a poor prognosis even if the tumor is diagnosed at an early stage. However, prompt and correct diagnosis still remains a challenge, especially in endemic areas with a high incidence of HCC. To date, HAC has only been reported in case series or single case reports. In order to get a clearer picture of this rare form of extrahepatic adenocarcinoma, analysis of the clinicopathological characteristics is necessary. We, herein, reported a case series using a database that included the clinicopathological features and the follow-up information from a total of 31 HACs in the stomach to give a brief view of this rare carcinoma in southwest region of China—the highest-risk region for gastric cancer.

## 2. Material and Methods

Thirty-one cases (0.39%) of gastric carcinoma were diagnosed as hepatoid adenocarcinoma (HAS) in West China Hospital, Sichuan University of China from January 2005 to December 2012. In order to offer a brief overview of the clinicopathological and prognostic characteristics, we collected the medical data of these cases to conduct a retrospective study.

The potential prognostic factors have been carefully recorded, including parameters related to the patient (sex, age, and AFP elevation), as well as the parameters of the tumor, including location, the TNM classification according to UICC (International Union Against Cancer)/AJCC (American Joint Committee on Cancer), histological characteristics, and immunohistochemistry presentations. Gross pathologic presentations under endoscopy of early gastric cancers were classified according to the criteria proposed by Japanese Society for Gastroenterology (JSG). For advanced gastric cancer, the Borrmann classification was applied.

Results were expressed as the mean ± SD and percentages. Statistical analysis was performed using SPSS 11.0 for Windows software (SPSS, Chicago, IL, USA). The clinicopathological factors were assessed by the Kaplan-Meier method and were compared by the Log-Rank test. Cox model was used for a multivariate analysis. Patients were followed up for at least three years after operation. All *P* values cited were two sided and <0.05 was judged to be statistically significant.

## 3. Results

### 3.1. Clinical Characteristics of HAC

Patients with HACs were usually male and the male to female ratio was 2.1 : 1 (21 versus 10). The average age of HAC patients was 51.2 ± 20.8 years, with a range of 32 to 87 years. Most patients entered the hospital with atypical symptoms of abdominal discomfort or pain, abdominal distention, anorexia, hematochezia, marasmus, and so on. A majority of patients had elevated serum AFP (87.1%) ranging from 37 ng/mL to 6400 ng/mL.

### 3.2. Endoscopic Pathologic Presentations of HAC

In our study, HACs were mostly derived from the body (38.7%), while only 29.0% and 32.3% rooted at antrum and eso-cardia, respectively. In total, 10 HACs in the early phases were separately demonstrated as protruded (type I, 5 cases), superficial elevated (type IIa, 1 case), superficial depressed (type IIc, 1 case), and excavated lesions (type III, 3 cases). There were 21 (71.0%) cases of advanced HACs (10 cases Borrmann type I, 6 cases Borrmann type II, 4 cases Borrmann type III, and 1 case Borrmann type IV lesions).

### 3.3. Metastasis of HAC

Imaging examinations of abdominal ultrasound, compute tomography scan, and magnetic resonance imaging were used in the assessment of metastasis and recurrence pre-, during, or post operation. Metastases from original organs were usually found once HACs were confirmed. 17 (54.8%) and 8 (25.8%) patients were confirmed to have lymphatic and distant metastasis separately before or at the surgery (including 6 hepatic metastases and 2 lung metastases). Liver and gastric tumors were often found synchronously, or liver tumors could be detected before gastric tumors. During at least three-year follow-up, the most common sites of metastasis were lymph nodes (77.4%), followed by liver (41.9%), lung (16.1%), peritoneum (6.5%), pancreas (6.5%), spleen (3.2%) and cerebral parenchyma (3.2%), metastasis. Only 7 (22.6%) patients survived without metastasis. The TNM stages from I to IV accounted for 22.6%, 19.4%, 32.3%, and 25.7%, respectively.

### 3.4. Pathological Characteristics of HAC

#### 3.4.1. Hematoxylin and Eosin Staining

The specimen mainly consisted of two histological components stained by hematoxylin and eosin. The predominant part was composed of large polygonal hepatocyte-like cells with clear and abundant eosinophilic cytoplasm, centrally located pleomorphic, and hyperchromatic nucleoli in some of the cells. Solid and tubular structures of poorly (54.8%) and highly (29.0%) differentiated adenocarcinoma were intermingled with hepatoid areas. Despite further detailed inspection with light microscopy, intravascular proliferation of tumor cells, intravenous carcinomatous thrombi, bile secretion, reticular patterns, and Schiller-Duval bodies were not observed. Intracytoplasmic hyaline globules stained by Periodic acid-Schiff (PAS) and resistant to diastase digestion were found occasionally (9.7%) in the hepatoid and adenocarcinoma cells. Hemorrhage and necrosis were seen in some areas of the tumor.

#### 3.4.2. Immunohistochemical Staining

Further immunohistochemistry stains were usually done for differential diagnosis. The majority of HACs exhibited positive AFP staining (90.3%) in the HCC-like cells, but positive Hep Par 1 stains were found in only 25.8% of HAC patients. Polyclonal carcinoembryonic antigen (pCEA) immune reactivity was found in 61.3% of HAC patients and mostly displayed in the poorly differentiated adenocarcinoma cells and in a large number of hepatoid cells. Among epithelial markers, all HACs (100%) exhibited CK18 and CK19 stains, while only a small proportion (35.5%) of HAC patients showed positive stains for CK20 and CK7 which were predominantly detected in the poorly differentiated adenocarcinoma cells. CD30 were negatively stained. High proportions of HAC tumors were positive for pancytokeratin (AE1/AE3) stains (77.4%). One HAC patient had specific staining for Cromogranin A (CgA) and CD56, which were focally positive in both adenocarcinoma and hepatoid areas, illustrating neuroendocrine carcinomatous features, while synaptophysin (SYN) was negative. Ki-67 expression in the tumor cell nuclei was moderately elevated (Ki-67 labeling index, 20–40%).

### 3.5. Survival and Influencing Factors

**Figure 1:**
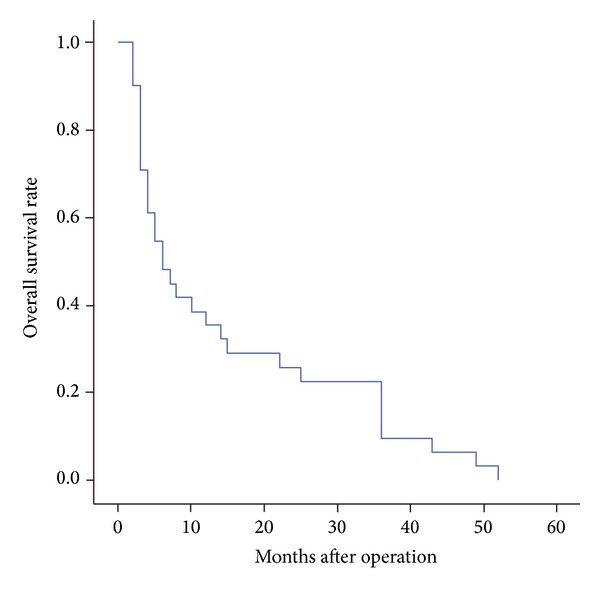
The overall survival of HACs.

Patients were followed up through telephone or clinical visits. The median follow-up time was 9 months. No patient was lost to follow up at the end of three years after operation. The three-year survival rate of those 31 patients was 22.6%. And the median overall survival time was 6 months. Results showed that TNM stages, immunohistochemical staining of AFP, CEA, CK7, and CK20 had statistically relation with the survival using the methods of Kaplan-Meier, Log-Rank test, and Cox multivariate regression analysis, *P* < 0.05 (Tables 1 and 2, Figure 1).

## 4. Discussion 

HAC is a rare but important special type of gastric adenocarcinoma, which was defined as a tumor composed of polygonal cells arranged in a solid or trabecular manner that resembles hepatocellular carcinoma, regardless of AFP production [1]. Reports have shown that HAC may only account for 0.17%–15% of all gastric carcinoma [2, 3]. Although the morbidity of HAS (0.39%) was still low in our hospital like another research from China (0.36%) [3], the poor prognosis was also quite astonishing in accordance with previous reports, shown by the high metastatic rate before surgery to the liver (19.4%) and lymph nodes (54.8%), large proportion of advanced HACs (71.0%) at admission, short median overall survival time (6 months), and low three-year survival rate (22.6%) [2–4]. Lack of clear recognition may lead to the misdiagnosis of HAS in the stomach. Therefore, it requires special attention to the differentiation.

The original description of gastric HAC was based on the morphological similarities to HCC and increased level of AFP in serum [5]. Further analysis of large amount of cases and the biological nature of AFP specified that the elevation of AFP is a unique but not essential feature and thus emphasized the histological characteristics similar to HCC, regardless of AFP production [1]. Since still 87.1% of HACs in the stomach exhibited elevated AFP levels, differential diagnoses were yet needed to be made from noncancerous conditions like cirrhosis and hepatitis. And in the ones with original liver HAC metastasis, other cancerous changes such as original HCC, hepatoblastoma, and germ cell tumors (especially yolk sac carcinoma), in addition the rare case of papillary or tubular gastric carcinoma type with higher serum AFP, should be ruled out [6]. Findings from the medical histories, imaging examinations, operations, and histology helped us differentiate our case.

In order to identify the high-risk population with HACs, Kaplan-Meier and Cox multivariate regression analysis were conducted. We find out that TNM stage was not surprisingly an independent risk factor like in other types of gastric cancer. By immunohistochemistry stains, special chemomediators including AFP, CEA, CK7 and CK20 were confirmed crucial for survival assessment. The molecular mechanisms of poor survival related with AFP stain (*P* = 0.037) remain obscure. AFP had a suppressive effect on lymphocyte transformation, therefore, was postulated to be adverse to tumor suppression [7]. Koide et al. [8] have observed that AFP-producing gastric cancer has high proliferative activity, weak apoptosis, and rich neovascularization. Positive CEA staining was closely related to poor survival (*P* = 0.008) in those cases with poorly differentiated adenocarcinoma cells, indicating that the CEA which lead to the malfunctions and biologic misbehavior in cancer cells might exert harmful influence on HAC in the stomach [9]. Among epithelial markers, CK18/ CK19 and AE1/AE3 were usually positive for HAC (100% and 77.4%, resp.). In the differential diagnosis between HAC and HCC, HAC is strongly suggested if CK18/C19, AE1/AE3 stains showed strong positive findings [10]. Though being a low proportion of CK20 and CK7 (35.5%), patients without CK20 and CK7 staining lived a longer life (*P* = 0.000). The relation of CK20 and CK7 to histology classification, nonideal mucin phenotype, or several tumor-related molecules of cancer cells may play a role [11, 12]. Of those HACs, special attention was given to one case with the presence of neuroendocrine differentiation (positive CgA and CD56). Although gastric adenocarcinomas frequently have neuroendocrine differentiation with a prevalence ranging from 1.7% to 34.3%, HAC with neuroendocrine differentiation has rarely been described. Although the influence of neuroendocrine differentiation to the survival of HAC remained obscure, researchers were inclined to regard it as disadvantageous factor to induce poor differentiation and metastasis [13]. Though being not identified as reproductive cells, neuroendocrine cells could induce mitogenic peptide which stimulated proliferation of circumambient tumor cells and aggravates tumor growth through paracrine [14]. Further surveillance of this rare case was ongoing and would be reported later.

Although the number of cases in our study was relatively small, the results could still provide some clinical value. HAC of the stomach, which was defined as a carcinoma histologically resembling HCC, was a rare type of adenocarcinoma with poor prognosis because of the aggressive progression. In order to gain a better prognosis, special attention to differentiation from cancerous or noncancerous conditions with AFP elevation should be paid. And early identification of undesirable TNM stages, positive staining of chemomediators including AFP, CEA, CK7, and CK20, which were all independent risk factors for HAS, could improve the assessment of high-risk patients for better intervention.

## Figures and Tables

**Table 1:** Prognostic factors related to prognosis by the Kaplan-Meier method.

Clinicopathological factors	Total number	Mean time (month)				*P* values
		Estimate	Std. error	95% CI	
				Lower	Upper
Gender						
Female	10	20.700	6.002	8.896	32.504	0.384
Male	21	12.000	2.963	6.192	17.808	
Elevation of serum AFP						
Yes	27	15.704	3.201	9.429	21.978	0.467
No	4	8.750	2.926	3.015	14.485	
Location of cancer						
Eso-cardia	10	16.800	5.083	6.837	26.763	0.975
Body	12	12.417	4.485	3.627	21.207	
Antrum	9	15.778	5.734	4.538	27.017	
pTNM stages						
I	7	36.714	5.838	25.272	48.157	0.000
II	6	20.333	3.853	12.782	27.885	
III	10	5.800	0.867	4.101	7.499	
IV	8	2.750	0.250	2.260	3.240	
AFP IHC						
Yes	28	14.679	2.902	8.991	20.366	0.037
No	3	37.000	13.503	0.000	42.466	
Hep Par 1 IHC						
Yes	8	8.625	4.931	0.000	18.291	0.105
No	23	16.957	3.354	10.383	23.530	
CEA IHC						
Yes	19	8.737	2.867	3.117	14.357	0.008
No	12	24.417	4.642	15.317	33.516	
CK20 IHC						
Yes	11	3.444	0.556	2.356	4.533	0.000
No	20	19.455	3.545	12.507	26.402	
CK7 IHC						
Yes	11	3.333	0.715	1.932	4.735	0.000
No	20	17.560	3.284	11.124	23.996	
AE1/AE3 IHC						
Yes	24	13.571	4.613	4.530	22.613	0.786
No	7	15.167	3.447	8.411	21.922	

**Table 2:** Prognostic factors related to prognosis by Cox multivariate regression analysis.

Clinicopathological factors	*P* values	RR	95% CI	
			Lower	Upper
pTNM stages	0.000	17.649	3.590	7.202
AFP IHC	0.023	0.371	0.173	1.263
CEA IHC	0.044	0.268	0.074	0.965
CK20 IHC	0.014	14.063	1.703	16.108
CK7 IHC	0.004	0.029	0.003	0.333
